# Jefferson Medical College, Philadelphia

**Published:** 1858-03

**Authors:** 


### Jefferson Medical College, Philadelphia.

Though we have not received the catalogue of the students of this Institution, we learn from a Philadelphia paper that it is in a flourishing condition, and is well sustained by numbers, (as well as by the talents of its able faculty,) notwithstanding the hard times. It has a list of 501 students, of whom 126 are from Pennsylvania; 74 from Virginia; 34, North Carolina; 30, Georgia; 25, Mississippi; 26, Kentucky; 25, Alabama; 18, South Carolina; 16, New Jersey; 14, Tennessee; 13, Ohio; 12, Indiana; 10, Maryland, and smaller numbers from other States. Of the whole number, 18 are from New England, 150 from New York, New Jersey and Pennsylvania, 279 from the Southern States, 36 from the free States of the West; or 204 from the North, 279 from the South.

As we receive reliable information, we shall take pleasure in recording the condition of other Institutions in the country.

